# Regulation of Development of CD56^bright^CD11c^+^ NK-like Cells with Helper Function by IL-18

**DOI:** 10.1371/journal.pone.0082586

**Published:** 2013-12-20

**Authors:** Wen Li, Akico Okuda, Hideyuki Yamamoto, Kyosuke Yamanishi, Nobuyuki Terada, Hiromichi Yamanishi, Yoshimasa Tanaka, Haruki Okamura

**Affiliations:** 1 Laboratory of Tumor Immunology and Cell Therapy, Hyogo College of Medicine, Hyogo, Japan; 2 Department of Pathology, Hyogo College of Medicine, Hyogo, Japan; 3 Department of Neuropsychiatry, Hyogo College of Medicine, Hyogo, Japan; 4 Hirakata General Hospital for Developmental Disorders, Hirakata, Osaka, Japan; 5 Center for Innovation in Immunoregulative Technology and Therapeutics, Graduate School of Medicine, Kyoto University, Kyoto, Japan; Centre de Recherche Public de la Santé (CRP-Santé), Luxembourg

## Abstract

Human γδ T cells augment host defense against tumors and infections, and might have a therapeutic potential in immunotherapy. However, mechanism of γδ T cell proliferation is unclear, and therefore it is difficult to prepare sufficient numbers of γδ T cells for clinical immunotherapy. Recently, natural killer (NK)-like CD56^bright^CD11c^+^ cells were shown to promote the proliferation of γδ T cells in an IL-18-dependent manner. In this study, we demonstrated that the NK-like CD56^bright^CD11c^+^ cells could directly interact with γδ T cells to promote their sustained expansion, while conventional dendritic cells (DCs), IFN-α-induced DCs, plasmacytoid DCs or monocytes did not. We also examined the cellular mechanism underlying the regulation of CD56^bright^CD11c^+^ cells. CD14^+^ monocytes pre-incubated with IL-2/IL-18 formed intensive interactions with CD56^int^CD11c^+^ cells to promote their differentiation to CD56^bright^CD11c^+^ cells with helper function. The development of CD56^bright^CD11c^+^ cells was suppressed in an IFN-α dependent manner. These results indicate that CD14^+^ monocytes pretreated with IL-2/IL-18, but neither DCs nor monocytes, play a determining role on the development and proliferation of CD56^bright^CD11c^+^ cells, which in turn modulate the expansion of γδ T cells. CD56^bright^CD11c^+^ NK-like cells may be a novel target for immunotherapy utilizing γδ T cells, by overcoming the limitation of γδ T cells proliferation.

## Introduction

Human γδ T cells recognize pathogens and autologous stress antigens and are involved in stress surveillance responses and maintenance of homeostasis in hosts [1], [2]. They belong to the innate immune system and regulate acquired immunity through cytokine production and antigen presentation [3]–[6]. Because γδ T cells distinguish infected cells and cancer cells from normal cells by detecting stress-induced molecules using γδ T cell receptors (TCRs) and natural killer (NK) cell receptors, stimulation of γδ T cells has gained attention as a potential therapeutic intervention for infections and malignancies [7]–[12]. However, cancer immunotherapy targeting γδ T cells has met with limited success because of the difficulty of inducing the expansion of γδ T cells in some cancer patients.

γδ T cells are effectively activated by small foreign and self metabolites such as (*E*)-4-hydroxy-3-methylbut-2-enyl diphosphate and isopentenyl diphosphate in a classical MHC and MHC-related molecule-independent manner [13], [14]. It is important to note, however, that CD14^+^ antigen-presenting cells are required for the recognition of metabolite antigens by γδ T cells, yet the precise mechanism for the recognition at the molecular level remains unclear [15]. In addition, the involvement of other immune cells such as NK cells and dendritic cells (DCs) in this recognition process has not been thoroughly explored [16]–[21].

It was previously demonstrated that human peripheral NK cells activated by *Mycobacterium tuberculosis* augmented the proliferation of γδ T cells [22]. Peripheral blood DCs expressing CD56, an NK cell marker, promoted Th1-type responses of γδ T cells stimulated by bisphosphonate and IL-2 [23]. We previously observed that CD56^bright^CD11c^+^ cells were involved in the IL-18-mediated expansion of γδ T cells stimulated by IL-2 and zoledronic acid (ZOL) [24], [25]. In addition, it was demonstrated that IL-18-induced NK cells exhibited helper functions in the development of cytotoxic T lymphocytes (CTLs), although whether these NK cells also acted on γδ T cells is yet to be determined [26], [27].

IL-18 was originally identified as an IFN-γ-inducing factor that activates NK cells [28]. Recent studies showed that IL-18 is produced by a wide variety of cells including non-immune as well as immune cells and the physiological roles of IL-18 extend far beyond serving merely as a cytokine inducer. For example, IL-18 is involved in angiogenesis [29] and metabolic syndromes [30], [31]. 

Therefore, it is necessary to determine the various functions of IL-18 to clarify its central, biological and pathophysiological roles. IL-18 is produced as an inactive precursor and converted to an active form by the catalytic action of the inflammasome, which is composed of NLRP3, ASC, and caspase-1. Because it is activated by various stresses such as oxidation [32], IL-18 is considered to be one of the stress-sensing molecules. As IL-18 activates intracellular signals related to cell viability in NK cells [33] and memory-type CD8^+^ T cells [34] it is likely that IL-18 promotes proliferation and differentiation of certain cells expressing IL-18 receptors through activation of survival signals.

It was previously reported that IFN-α promoted the differentiation of monocytes to IFN-α-DCs that promote the generation of CD8^+^ CTLs, in addition to its anti-viral properties [35]–[37]. Several studies also indicated that IFN-α might activate γδ T cells during infection [38]–[40]. In the present study, we examined how the development and proliferation of novel NK-like CD56^bright^CD11c^+^ cells were differentially regulated by CD14^+^ monocytes under the influence of IL-2/IL-18 or other cytokines including IFN-α, which will hopefully contribute to our understanding of the mechanisms behind the efficient expansion of human γδ T cells.

## Materials and Methods

### Reagents

Recombinant human IL-18 and ZOL were kindly provided by GlaxoSmithKline plc (Research Triangle Park, NC) and Novartis AG (Basel, Switzerland), respectively. We synthesized 2-Methyl-3-butenyl-1-diphosphate (2M3B1PP) as described previously (25). GM-CSF, IL-2, IL-4, TNF-α, IFN-α, anti-IL-18Rα monoclonal antibody (mAb, clone: 70625.1111) were purchased from R&D Systems Inc. (Minneapolis, MN). Human AB serum was purchased from GemCell™ (Gemini, Bio-Products, West Sacramento, CA). All of the dye-conjugated mAbs were purchased from BD PharMingen (San Jose, CA) and BioLegend (San Diego, CA): CD3 (Clone: HIT3a), αβ-TCR (Clone: IP26), γδ-TCR (Cat: 555716), Vδ2 (Cat: 555738), CD11a (Clone: HI111), CD11c (Clone: 3.9), CD16 (Clone: 3G8), CD18a (Clone: TS1/18), CD25 (Clone: MEM-181), CD28 (Clone: CD28.2), CD40 (Clone:HB14), CD40L (Clone: 24-31), CD54 (Clone: MEM-111), CD56 (Clone: MEM-188), CD62L (Clone: DREG-56), CD80 (Clone: 2D10), CD83 (Clone: HB15e), CD86 (Clone: IT2.2), CD122 (Cat: 554522), CD123 (Clone: 6H6), CD209 (DC SIGN, Clone: DCS-8C1), HLA-ABC (Clone: W6/32) HLA-DR (Clone: L243), CCR5 (Clone: T21/8) and CCR7 (Cat: 552174). CD14^+^ beads, CD56^+^ beads, CD303^+^ (BDCA-2, Cat: 130-090-691) beads, a Plasmacytoid Dendritic Cell Isolation kit (130-092-207) and a TCR γδ^+^ T cells Isolation kit (130-092-892) were purchased from Miltenyi Biotec Inc. (Auburn, CA). Human monocyte enrichment kit was obtained from Stemcell Technologies Inc. (Vancouver, BC, Canada). CellTrace™ CFSE Cell Proliferation kit (C34554) was purchased from Molecular Probes, Invitrogen (Eugene, Oregon).

### Cell separation and culture

Peripheral blood mononuclear cells (PBMCs) were purified from heparinized blood of healthy human donors (ranging from 25 to 40 years of age) by Ficoll-Hypaque gradient centrifugation (GE Healthcare Bio-Sciences AB, Uppsala, Sweden) after receiving institutional review board approval (The Ethics Review Board of Hyogo College of Medicine, No.1033–2011) and written informed consent. PBMCs were cultured in AlyS505N-O medium (Iscove's MEM-based serum-free medium, Cell Science & Technology Institute, Inc., Sendai, Miyagi, Japan) supplemented with streptomycin, penicillin, glutamate, and 5% AB serum at 37°C in a humidified atmosphere containing 5% CO_2_. Depletion of CD3^+^, CD56^+^, or CD14^+^ cells from PBMCs was conducted using LD columns and microbeads conjugated with their specific mAbs (Miltenyi Biotec Inc.). Purification of γδ T cells, CD56^+^, CD14^+^, and plasmacytoid DC (pDC) cells was carried out by positive selection using MS columns (Miltenyi Biotec Inc.). Cell isolation was performed according to the manufacturer's instructions. Growth factors, stimulators, and inhibitors were added to cultures at the following concentrations: 1 μM ZOL, 100 μM for 2M3B1PP, 10 ng/ml for IL-2, 100 ng/ml for IL-18, 5 ng/ml for GM-CSF, 20 ng/ml for IL-4, 1,000 U/ml for IFN-α, and 2.5 μg/ml for anti-IL-18Rα mAb, unless otherwise noted. Cell viability was determined by trypan blue dye exclusion or propidium iodide staining, and the cell number was calculated based on flow cytometry and cell viability. Cellular surface markers were analyzed using a FACSCalibur flow cytometer (Becton Dickinson, Franklin Lakes, NJ). CD14^+^, CD56^int^CD11c^+^, CD56^int^CD11c^−^, CD56^bright^CD11c^+^, and CD56^bright^CD11c^−^ cells were purified using a FACSAria cell sorter (Becton Dickinson) according to the manufacturer's instructions.

### Expansion of γδ T cells and CD56^bright^CD11c^+^ cells

For the expansion of γδ T cells, PBMCs were stimulated with ZOL/IL-2/IL-18 at 1–5×10^5^ cells/well in a 48-well plate. CD56^bright^CD11c^+^ cells were expanded in culture of PBMCs or CD3^+^ T cell-depleted PBMCs at 1–5×10^5^ cells/well in a 48-well plate in the presence of IL-2/IL-18.

### Preparation of DCs

For preparation of DCs, purified monocytes were incubated with GM-CSF/IFN-α or GM-CSF/IL-4 for 3–4 days in a 24-well plate at a cell concentration of 5×10^5^/well. The resulting DCs (IFN-DCs or IL-4-DCs) were examined for the expression of CD56, CD11c, CD14, and CD62L. pDCs were purified (>95%, defined by flow cytometry) via positive selection with a CD303^+^ pDC Isolation kit.

### Flow cytometry

Cells were stained with FITC-, PE-, APC-, or biotin-conjugated mAbs specific for CD3, γδ TCR, Vδ2, CD11c, CD11a, CD18a, CD14, CD16, CD25, CD28, CD40, CD40L, CD54, CD56, CD62L, CD80, CD83, CD86, CD122, CD123, CD209, CD303, CCR5, CCR7, HLA-ABC HLA-DR, IL-18Rα or IL-18Rβ, for 20 min at 4°C and then analyzed by a FACSCalibur flow cytometer. Human AB serum (10%) was used as an Fc receptor blocker and mouse immunoglobulins (Igs, BD Pharmingen) were used as isotype controls. Data were analyzed by CellQuest software (BD Biosciences, San Jose, CA).

### Cell morphology

Cells were cultured in 24-well plates (Corning Inc., Corning, NY) and observed under a Nikon Digital Sight-Mac6000 microscope. The images were analyzed using a Soft Lumina Vision analyzer.

### Confocal microscopic analysis

PBMCs derived from a donor whose initial Vδ2^+^ T cell frequency was 12.9% were incubated in the presence of ZOL/IL-2 for 10 days. The resulting cells (Vδ2^+^ T cells >99%) were stained with CellTracker™Green CMFDA (Life Technologies Corp., Carlsbad, CA) according to the manufacturer's protocol. For preparation of CD56^bright^CD11c^+^ cells, PBMCs were incubated with IL-2/IL-18 for 4 days and CD3^+^ T cells were removed using a CD3 Isolation kit and an AutoMACS Pro cell separator (Miltenyi Biotec Inc.). The CD3^−^ cells were incubated for an additional 5 days in the presence of IL-2 and IL-18 and CD3^+^ T cells were removed. After incubation for an additional day, the CD56^bright^CD11c^+^ cells were harvested and stained with CellTracker™Red CMTPX (Life Technologies Corp.) according to the manufacturer's protocol. The green- and red-stained cells were mixed and placed in 35-mm glass base dishes (Glass 27φ) (Asahi Glass Co., Ltd., Tokyo, Japan). After incubation with or without ZOL for 4 h, the cells were observed under an LSM 710 Laser Scanning Microscope (Carl Zeiss AG, Oberkochen, Germany) and the images were analyzed using Zen software (Carl Zeiss AG).

### Cell Division Assay

CD56^+^ cells were enriched from PBMCs by depleting CD3^+^ and CD14^+^ cells. Monocytes were purified using a human monocyte enrichment kit. The enriched CD56^+^ cells were gently mixed with 3 μM carboxyfluorescein diacetate, succinimidyl ester (5(6)-CFSE; molecular probe C-1157), and allowed to stand at room temperature for 15 min. An equal volume of 100% fetal bovine serum was added and incubated for 15 min at 37°C to remove excess CFSE. Labeled CD56^+^ cells and purified CD14^+^ monocytes were mixed at a ratio of 1∶1 at a cell density of 2×10^5^/well, incubated for 4 days, and then analyzed for the expression of CFSE, CD56, and CD11c.

### Transwell test

Transwell test was performed using Costar transwell chambers with a pore size of 5 μm for cell penetration tests, and a pore size of 0.4 μm for soluble factor permeability tests. Purified CD14^+^ monocytes (>98% purity) were placed in the lower chambers and CD56^+^ cells (>95% purity) in upper chambers. After incubation for 5 days, the number of cells in the lower chambers was counted and the surface expression of CD14, CD56, and CD11c was analyzed by flow cytometry.

### Statistical analysis

Statistical analysis was performed using Student's *t*-test or Bonferroni multiple comparisons test and expressed as the means ± SD. Values of * p<0.05, or **p<0.01 were considered statistically significant.

## Results

### Comparison of IL-18-induced CD56^bright^CD11c^+^ cells and conventional DCs on the helper function on γδ T cells

Although we previously demonstrated that IL-2/IL-18-induced CD56^bright^CD11c^+^ cells enhanced ZOL-mediated expansion of γδ T cells, the mechanism underlying the regulation of these cells in the culture of PBMCs with ZOL/IL-2/IL-18 has not been fully clarified. Consistent with our previous report, 1∼4% and approximately 2% of freshly isolated PBMCs exhibited CD56^int^CD11c^+^ and Vδ2^+^ phenotypes, respectively ([25], data not shown). When PBMCs were incubated in the presence of ZOL/IL-2/IL-18 for 10 days, the number of CD56^int^CD11c^+^ cells was decreased in the CD3^−^ gated area. In contrast, CD56^bright^CD11c^+^ cells appeared and eventually represented 10∼18% of the total cells in the culture [25]. Whereas CD56^bright^CD11c^+^ cells were CD80/86^high^, NKG2D^high^, and HLA-DR^high^, CD56^int^CD11c^+^ cells were CD80/86^low^, NKG2D^low^, and HLA-DR^low^, as previously reported [25]. On day 15, however, the intensity of CD11c on these cells was reduced and CD56^bright^CD11c^−^ cells formed the majority phenotype found among CD3^−^ cells (data not shown).

**Figure 1:**
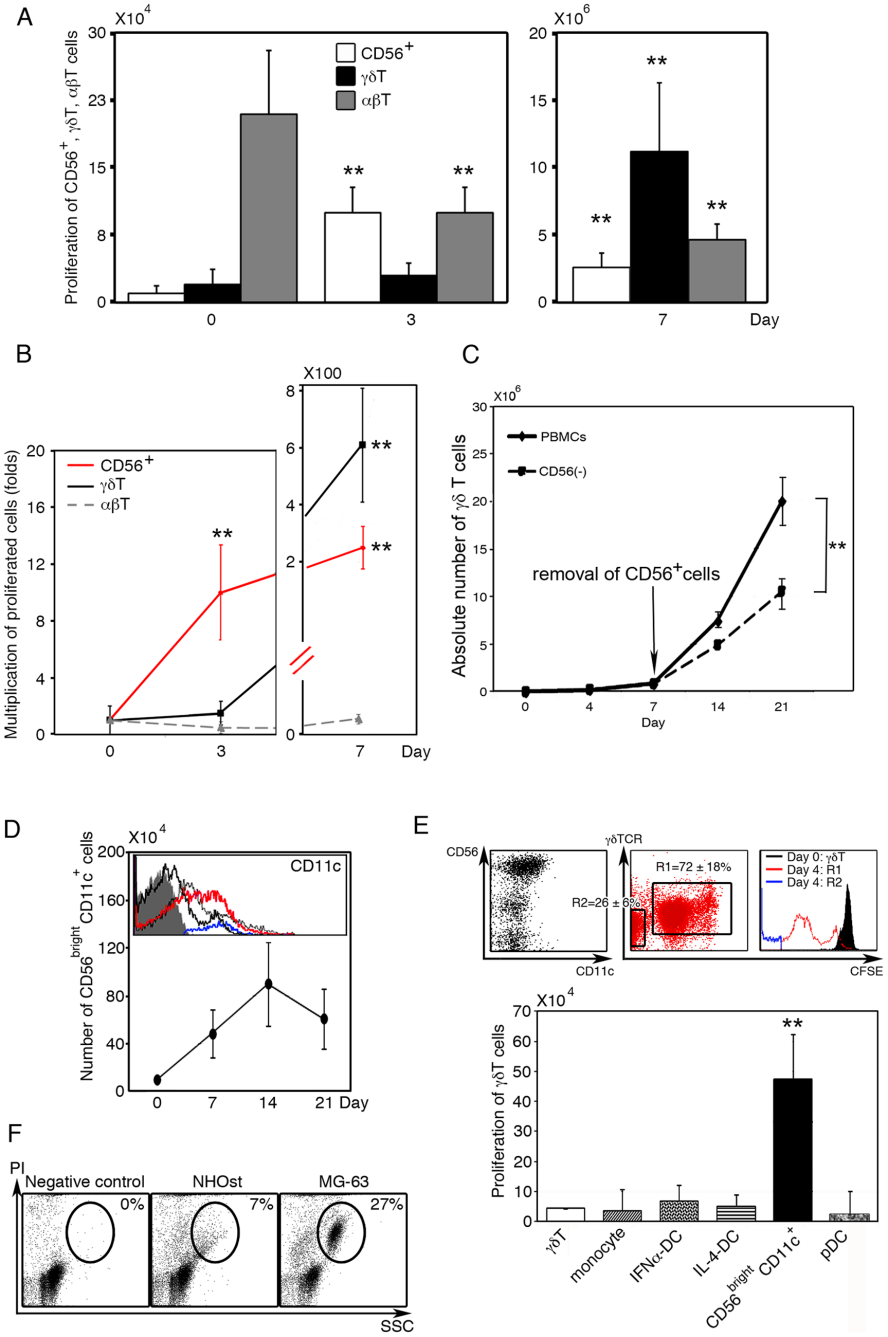
Development, change of phenotype and function of CD56^bright^CD11c^+^ cells induced by IL-18. (A) Time course of the development of CD56^bright^CD11c^+^ cells. PBMCs (1×10^5^ cells/0.5 ml/well) were stimulated with ZOL/IL-2/IL-18. The absolute numbers of CD56^+^ or CD56^bright^CD11c^+^ cells (white bar), γδ T cells (black bar) and αβ T cells (gray bar) were determined by flow cytometry and trypan blue dye exclusion test at day 0, day 3 (left), and day 7 (right). Data show mean ± SD (n = 10), **p<0.01. (B) Proliferation of CD56^+^ or CD56^bright^CD11c^+^ cells (red line), γδ T cells (black line), and αβ T cells (gray line). Data show mean ± SD (n = 10), **p<0.01. (C) Requirement of CD56^bright^CD11c^+^ cells for maximal sustained proliferation of γδ T cells. PBMCs were pre-stimulated with ZOL/IL-2/IL-18, and harvested on day 7. The proliferating cells were divided into 2 groups: one group was incubated with anti-CD56 antibody-conjugated beads and CD56^+^ cells were selectively removed. The other group was incubated with mouse IgG1-conjugated beads and used as a control. Both groups were re-incubated with ZOL/IL-2/IL-18 for another 14 days. Data show mean ± SD (n = 5), **p<0.01. (D) Development of CD56^bright^CD11c^+^cells and gradual reduced expression of CD11c. The number and CD11c expression of proliferated cells were analyzed by flow cytometry during the culture of CD3^+^ T cell-depleted PBMCs. (Grey shadow: isotype control; blue: CD56^int^CD11c^+^cells on day 0; red: CD56^bright^CD11c^+^cells on day 7; thin black line: day 14; and bold black line: day 21). Data show mean ± SD (n = 5). A histogram shown is a representative of five independent experiments. (E) Comparison among CD56^bright^CD11c^+^ cells, monocytes, and several subsets of DCs in helper activity for γδ T cells proliferation. Freshly isolated γδ T cells(5×104/well) were labeled with CFSE and co-cultured for 7 days with fresh CD14^+^ monocytes, IFN-α-DCs, IL-4-DCs, CD56^bright^CD11c^+^, or pDCs, at a ratio of 1∶1 in the presence of ZOL/IL-2/IL-18. Then, proliferative responses of γδ T cells were analyzed based on flow cytometry and trypan blue dye exclusion test. R1: γδ T cells labeled with CFSE undergoing cell division; R2: Unlabeled CD56^bright^CD11c^+^ cells. Data show mean ± SD (n = 5), **p<0.01. The result is representative of five independent experiments. (F) Differential cytotoxicity of CD56^bright^CD11c^+^ cells against normal osteoblast cells (NHOst) and tumor cells (MG-63). The cytotoxicity was assessed using standard propidium iodide staining. Dot plots shown are representative of three independent experiments.

Whereas both CD56^bright^CD11c^+^ cells and γδ T cells markedly proliferated in response to IL-2/IL-18/ZOL, the expansion of γδ T cells was preceded by the development and proliferation of CD56^bright^CD11c^+^ cells in terms of absolute number (Fig. 1A). Consistent with previous findings, the majority of freshly isolated PBMCs were αβ T cells, while CD56^int^CD11c^+^ and γδ T cells constituted a minor population (Fig. 1A; left). In addition, a small number of CD14^+^CD11c^+^ monocytes and CD56^+^CD11c^−^ NK cells were also present (data not shown). Stimulation of PBMCs by IL-2/IL-18/ZOL resulted in the development and expansion of CD56^bright^CD11c^+^ cells by around day 3 and they gradually increased the number thereafter. In contrast, γδ T cells began to proliferate at around day 4 and their absolute number and proportion surpassed those of CD56^bright^CD11c^+^ cells by day 7 (Fig. 1B). It is worthy of note that the frequency of CD56^bright^CD11c^+^ cells was almost negligible in freshly isolated PBMCs. CD56^bright^CD11c^+^ cells were, however, multiplied up to nearly 10-fold and 200-fold the initial number of CD56^int^CD11c^+^ cells, a putative precursor of CD56^bright^CD11c^+^ cells, at day 3 and day 7, respectively. On the other hand, γδ T cells apparently did not increase in number until day 3 or 4 at which point they rapidly increased and multiplied about 500-fold by day 7 (Fig. 1B). Although freshly prepared PBMCs contained αβ T cells as a major population, the number was declined gradually, with CD4^+^αβ T cells being barely detectable by day 7 in particular (data not shown). CD14^+^CD11c^+^ monocytes, which initially constituted 5–10% of PBMCs, completely disappeared by day 7 (data not shown).

As previously reported, when CD14^+^, CD56^+^, or CD11c^+^ cells were removed at the beginning of cell culture, the expansion of γδ T cells in PBMC cultures was significantly impaired even in the presence of IL-2/IL-18 [25]. Co-culture of IL-2/IL-18-induced CD56^bright^CD11c^+^ cells with freshly isolated γδ T cells, on the contrary, resulted in efficient expansion of γδ T cells in the presence of ZOL/IL-2/IL-18 [25]. Thus, CD56^bright^CD11c^+^ cells are a prerequisite for the efficient expansion of γδ T cells by day 7 of culture. In the present study, CD56^bright^CD11c^+^ cells were further depleted from ZOL/IL-2/IL-18-treated PBMCs at day 7 of culture by CD56 negative selection. The culture process of the resulting cells was re-started in the presence of ZOL/IL-2/IL-18 for another 14 days, and compared with that of consecutively cultured γδ T cells. As shown in Fig. 1C, proliferation of γδ T cells was significantly suppressed by removal of CD56^+^ cells although sustained partly. Thus, it was suggested that CD56^bright^CD11c^+^ cells were not continually essential during or after the logarithmic phase of cell expansion. However, the proficient supporting function of CD56^bright^CD11c^+^ cells in the early stage of culture was important for the maximal proliferation of γδ T cells (Fig. 1C).

The generation of CD56^bright^CD11c^+^ cells appeared to be independent of CD3^+^ T cells, as they developed in the culture of CD3^+^ T cell-depleted PBMCs supplemented with IL-2 and IL-18 (Fig. 1D). Disappearance of CD11c was confirmed by flow cytometry during the late stage of culture period (by day 21). The number of CD56^int^CD11c^+^cells, a possible precursor of CD56^bright^CD11c^+^cells, were present at the frequency of 4–5% in the initial culture, disappeared from the culture soon after the beginning of culture (data not shown). In the absence of T cells, CD56^bright^CD11c^+^ cells progressively increased in both frequency and absolute number up to day 14. Thereafter, the intensity of CD11c levels on CD56^bright^CD11c^+^ cells progressively decreased during the culture (Fig. 1D).

We next examined whether CD56^bright^CD11c^+^ cells generated in the culture of T cell-depleted PBMCs were able to help development and expansion of γδ T cells, comparing with various subsets of DCs or CD14^+^ monocytes. Freshly isolated CD14^+^ monocytes (>98%, purified by positive selection using MS column) were stimulated with IL-4/GM-CSF, or IFN-α/GM-CSF to induce IL-4-DCs or IFN-α-DCs. CD56^bright^CD11c^+^ cells were generated from the mixed culture of CD14^+^ monocytes and CD56^int^CD11c^+^ cells (selectively collected by FACS Aria cell sorter) in the presence of IL-2/IL-18, and CD303^+^ pDCs were purified by positive selection using MS columns. As shown in Fig. 1E, CD56^bright^CD11c^+^ cells strongly promoted the expansion of freshly isolated γδ T cells in the presence of ZOL/IL-2/IL-18. In contrast, monocytes, IFN-α/GM-CSF-induced DCs, IL-4/GM-CSF-induced DCs, and CD303^+^ pDCs failed to support the proliferation of γδ T cells (Fig. 1E).

Because CD56^bright^CD11c^+^ cells exhibited NK cell-like phenotypes, cytotoxic activity was determined by flow cytometric analyses. CD56^bright^CD11c^+^ cells (1×10^5^ cells/0.2 ml) were incubated with either NHOst osteoblastic cells or MG-63 osteosarcoma cells (1×10^4^ cells/0.2 ml) for 3 h. The cytotoxicity was assessed using standard propidium iodide staining. As shown in Fig. 1F, CD56^bright^CD11c^+^ cells preferentially lysed MG63 human osteosarcoma cells (27%), but not NHOst normal osteoblastic cells (7%). In addition, CD56^bright^CD11c^+^ cells killed K562 human erythrocytoma cells (data not shown). This demonstrates that CD56^bright^CD11c^+^ cells can distinguish tumor cells from normal cells and specifically kill the former cells.

### Interaction between γδ T cells and CD56^bright^CD11c^+^ cells

**Figure 2:**
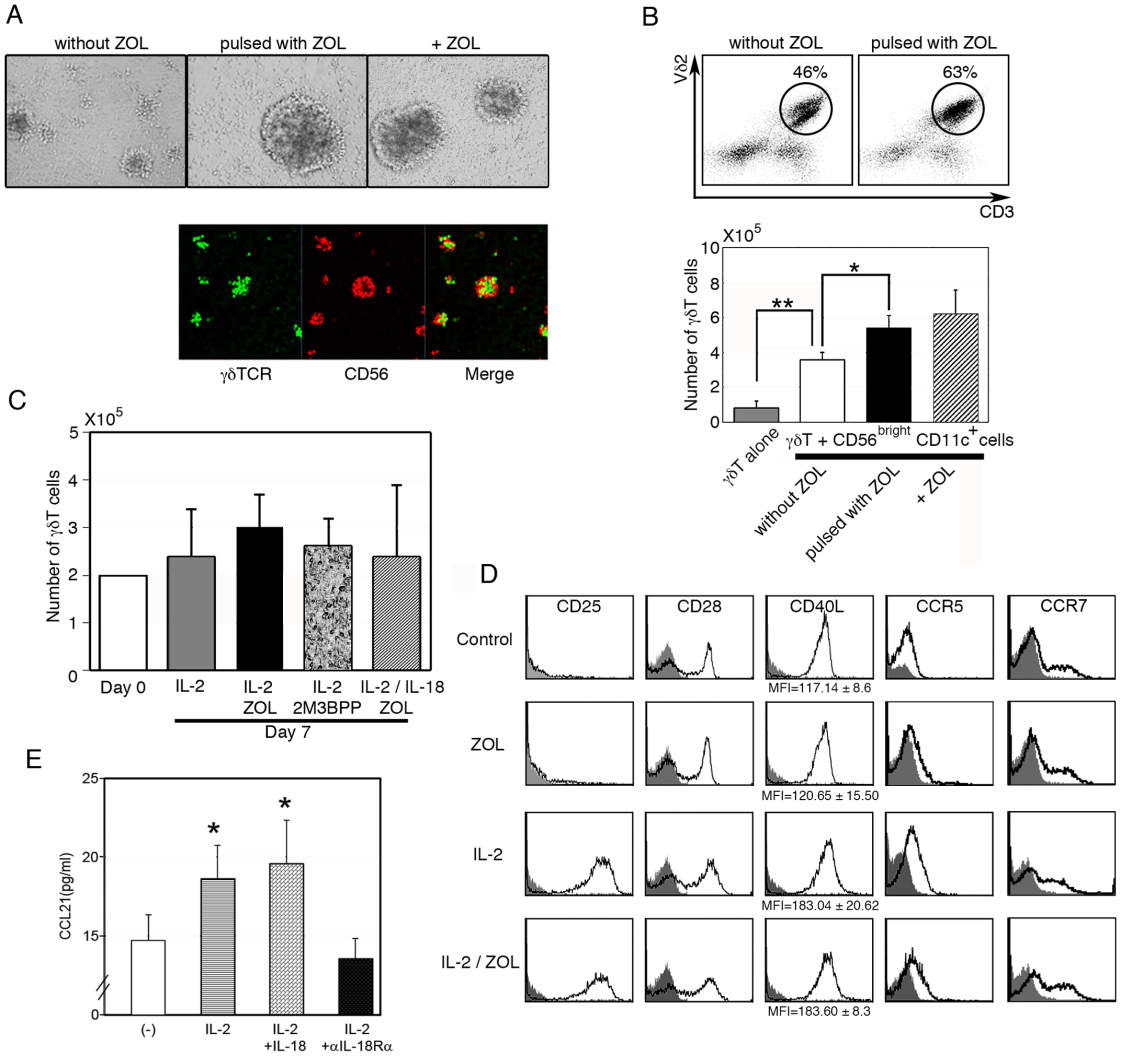
Interaction between γδ T cells and CD56^bright^CD11c^+^cells. (A) Cell aggregates observed in culture of γδ T cells mixed with CD56^bright^CD11c^+^ cells without pulse (left), pulsed with ZOL (center), and further incubated with ZOL (right) (upper panels) and confocal microscopic observation of the culture of γδ T cells (green) and CD56^bright^CD11c^+^ cells (red) in the presence of ZOL (lower panels). Data shown are representative of three independent experiments. (B) Flow cytometric analysis of γδ T cells incubated with CD56^bright^CD11c+ cells (upper panels) and the numbers of γδ T cells after expansion for 7 days (lower panel). Dot plots shown are representative of three independent experiments, and data of cell counts show mean ± SD (n = 5), *p<0.05, **p<0.01. (C) There was no sustained proliferation of freshly isolated γδ T cells in the absence of accessory cells even after stimulation with IL-2, IL-2/ZOL, IL-2/IL-18/ZOL, or IL-2/2M3B1PP for 7 days. Data show the mean ± SD (n = 5). (D) Expression of co-stimulatory molecules and chemokine receptors on γδ T cells after culture with ZOL, IL-2, or ZOL/IL-2 for day 7. The result of flow cytometric analysis is representative of three independent experiments. (E) Induction of CCL21 by IL-18 in CD56^bright^CD11c^+^cells. CCL21 concentration in the culture supernatant at 7 days was measured by ELISA. Data show mean ± SD (n = 5), *p<0.05.

Purified γδ T cells (more than 98% pure) were incubated with CD56^bright^CD11c^+^ cells (Fig. 2A, upper panel, left), ZOL-pulsed CD56^bright^CD11c^+^ cells (center), or ZOL-pulsed CD56^bright^CD11c^+^ cells with ZOL (right), and the cell cultures were observed under an optical microscope. ZOL-pulsed CD56^bright^CD11c^+^ cells formed larger aggregates than those without pulsing. To determine whether γδ T cells directly interacted with CD56^bright^CD11c^+^ cells in the cell aggregates, both cells were separately stained with either green or red fluorescent dye respectively and observed under a confocal microscopy after incubation in the presence of ZOL (Fig. 2A, lower panel). γδ T cells (green) and CD56^bright^CD11c^+^ cells (red) formed aggregates together and appeared to interact with each other. Similar results were obtained in the absence of ZOL (data not shown).

We next examined the effect of ZOL on the regulation of γδ T cell proliferation by CD56^bright^CD11c^+^ cells. Enriched CD56^bright^CD11c^+^ cells (1×10^5^/0.5 ml/well) prepared from CD3^+^ cells-depleted PBMCs were pulsed with or without ZOL for 2 hours before co-culture with purified γδ T cells (1×10^5^/0.5 ml/well). As shown in Fig. 2B, γδ T cells alone slightly reduced their number during culture (from 1×10^5^ to 0.7±0.42×10^5^), and CD56^bright^CD11c^+^ cells strongly promoted expansion of γδ T cells (from 1×10^5^ to 3.6±0.44×10^5^). Pulsing of CD56^bright^CD11c^+^ cells with ZOL significantly enhanced their helper function in proliferation of γδ T cells, both in percentage (without pulsing:46%; with pulsing:63%) and in absolute number (without pulsing: 3.6±0.44×10^5^; with pulsing: 5.42±0.69×10^5^). It was also shown that antigenic stimulation by ZOL could rather augment γδ T cell expansion (6.25±1.33×10^5^). Because freshly isolated γδ T cells failed to proliferate significantly in response to IL-2, IL-2/ZOL, or IL-2/2M3B1PP in the absence of accessory cells, even when IL-18 was present in the culture (Fig. 2C), it was suggested that IL-18-induced CD56^bright^CD11c^+^ cells played essential roles in the efficient expansion of γδ T cells.

Although stimulation of purified γδ T cells with either ZOL or 2M3B1PP alone did not alter the expression of co-stimulatory molecules such as CD28 and CD40L, addition of IL-2 either induced or augmented the expression of co-stimulatory molecules, CD28 and CD40L, and chemokine receptors, CCR5 and CCR7(Fig. 2D). CD56^bright^CD11c^+^ cells produced CCL21, a chemokine that binds to CCR7, in an IL-2/IL-18-dependent manner (Fig. 2E). These results strongly suggest that IL-2/IL-18 may facilitate the interaction between γδ T cells and CD56^bright^CD11c^+^ cells through co-stimulatory ligands/receptors, chemokine molecules/receptors, and adhesion ligands/receptors, leading to large cell aggregates and enhanced proliferation of γδ T cells.

### Requirement of IL-18-induced CD14^+^ monocytes for the development of CD56^bright^CD11c^+^ cells

**Figure 3:**
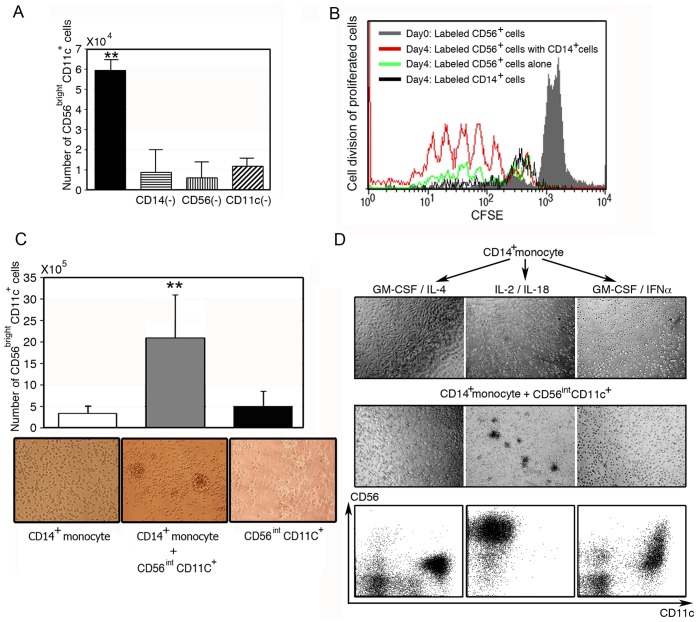
Role of CD14^+^ monocytes in the development of CD56^bright^CD11c^+^ cells. (A) CD14^+^, CD56^+^, and CD11c^+^ cells were required for the development of CD56^bright^CD11c^+^ cells. CD14^+^, CD56^+^, or CD11c^+^ cells were depleted from T cell-depleted PBMCs (2×10^4^ cells/0.5 ml) and stimulated with IL-2/ IL-18 for 7 days. The number of CD56^bright^CD11c^+^ cells was counted based on trypan blue dye exclusion. Data show mean ± SD (n = 5), **p<0.01. (B) Effect of CD14^+^ monocytes on the division of CD56^+^ cells. CFSE-labeled CD56^+^ cells were cultured with purified CD14^+^ monocytes at a ratio of 1:1 at a cell density of 2×105/well. After co-culture for 4 days, proliferating cells were analyzed by CFSE, CD56 and CD11c expression. A representative result of three independent experiments is shown. Division of CFSE-labeled CD14^+^ cells is also shown (black line). (C) Formation of large aggregates of CD56^bright^CD11c^+^ cells in the co-culture of CD56^int^CD11c^+^ cells and CD14^+^ monocytes in the presence of IL-2/IL-18 for 7 days, not in cultures of individual cell types. Data show mean ± SD (n = 4), **p<0.01, and the morphological data are representative of three independent experiments. (D) IL-2/IL-18-dependent cell aggregation in culture of CD14^+^ monocytes and CD56^int^CD11c^+^ cells. IL-4-DCs, IL-18-induced CD14^+^ monocytes and IFN-α-DCs were co-cultured with freshly isolated CD56^int^CD11c^+^ cells (1×105 cells/well) at a ratio of 1:1, respectively. After 12 h incubation, cell aggregates were observed under a microscope. Flow cytometric analyses are carried out after 3 days of co-culture. A representative result of three independent experiments is shown.

Next, we determined which types of cells were responsible for the development and expansion of CD56^bright^CD11c^+^ cells. CD14^+^, CD56^+^, or CD11c^+^ cells were further removed from CD3^+^ T cell-depleted PBMCs, and the resulting cells were stimulated with IL-2/IL-18 for 7 days. The development of CD56^bright^CD11c^+^ cells in the culture was significantly impaired by the removal of these cells (Fig. 3A). In addition, cell division analyses using CFSE-labeling further suggested that CD56^bright^CD11c^+^ cells were derived from CD56^+^ cells and vigorously proliferated during cell culture (Fig. 3B). In contrast, CFSE-labeled CD14^+^ cells failed to proliferate. In addition, IL-2/IL-18 failed to develop CD56^bright^CD11c^+^ cells in the culture of freshly isolated CD14^+^ monocytes (>98%, purified by positive selection using MS column) or CD56^int^CD11c^+^ cells alone (selectively collected by FACs Aria cell sorter), whereas CD56^bright^CD11c^+^ cells were generated in the mixed culture of CD14^+^ monocytes and CD56^int^CD11c^+^ cells in the presence of IL-2/IL-18, resulting in extensive cell aggregation (Fig. 3C). Next, the effect of various cytokines on CD14^+^ monocytes in the generation of CD56^bright^CD11c^+^ cells was examined (Fig. 3D). Freshly isolated CD14^+^ cells were pretreated with GM-CSF/IL-4, IL-2/IL-18, and GM-CSF/IFN-α for 3 days to induce IL-4-DCs, IL-18-primed CD14^+^ monocytes, or IFN-α-DCs, respectively. Then, freshly isolated CD56^int^CD11c^+^ cells were added to the cultures (1×10^5^cells/well) at a ratio of 1:1. After 12 h incubation, cells were observed under a microscope. As shown in Fig. 3D, cell aggregates appeared in the co-culture of CD14^+^ monocytes pretreated with IL-2/IL-18 and CD56^int^CD11c^+^ cells, but GM-CSF/IL-4-induced conventional DCs or GM-CSF/IFN-α-induced DCs failed to form cell aggregates when cultured with CD56^int^CD11c^+^ cells. Flow cytometric analyses of the expanded cells were carried out after 3 days of co-culture. There was a marked difference in the expression of CD11c and CD56 among cells. This suggested that stimulation of CD14^+^ monocytes by IL-2/IL-18 was essential for the development of CD56^bright^CD11c^+^ cells from CD56^int^CD11c^+^ cells (Fig. 3C and D).

### Effect of IL-18 on CD14^+^ monocytes involved in the development of CD56^bright^CD11c^+^ cells

**Figure 4:**
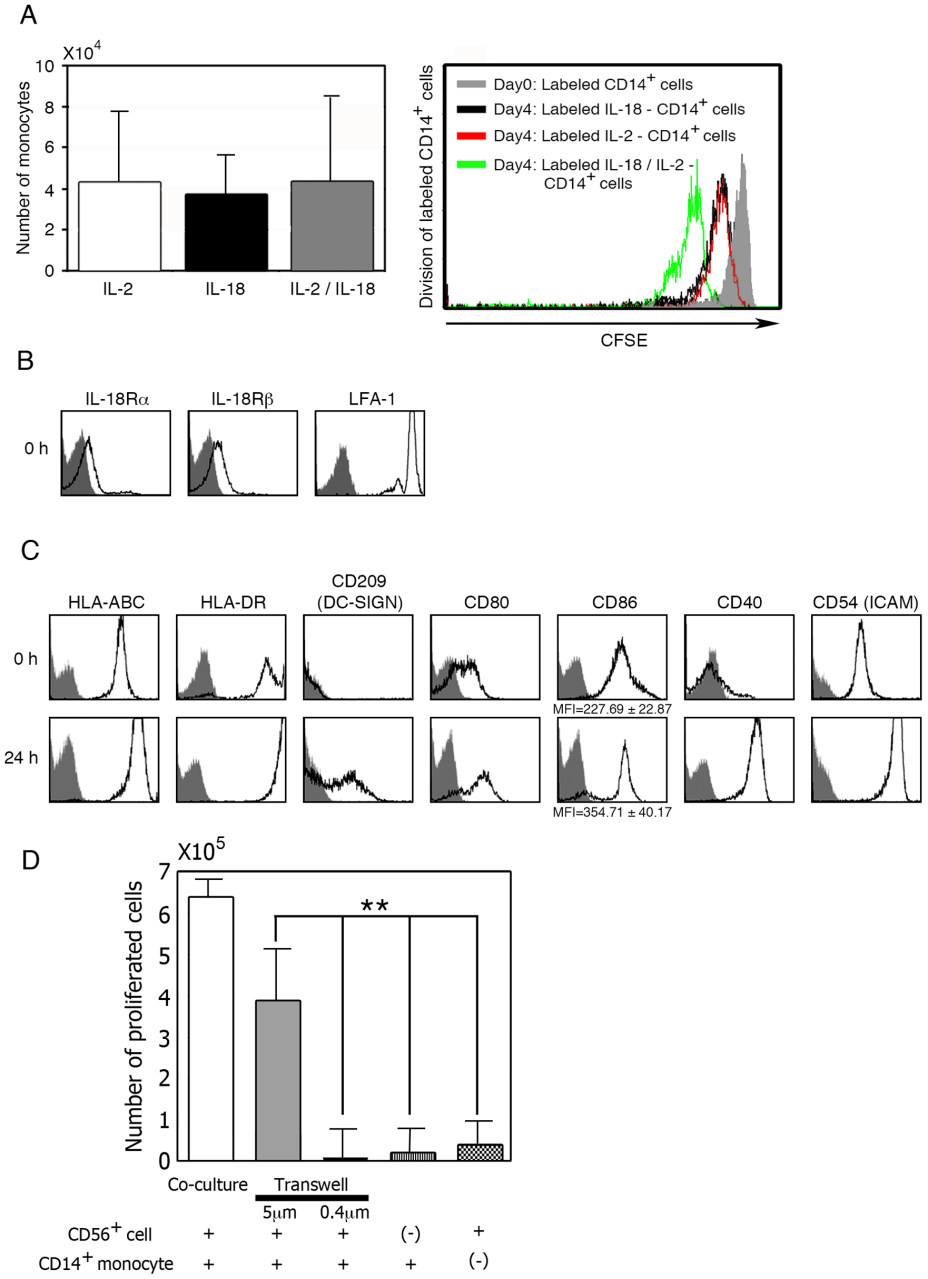
Phenotypic and functional analyses of CD14^+^ monocytes involved in the generation of CD56^bright^CD11c^+^ cells. (A) Effect of IL-2/IL-18 on CD14^+^ monocytes proliferation. Data show mean ± SD (n = 4). (B) Analysis of IL-18 receptors and LFA-1 expressed by fresh CD14^+^ monocytes by flow cytometry. A representative result of three independent experiments is shown. (C) Flow cytometric analysis of CD14^+^ monocytes stimulated with IL-2/IL-18 after 24h culture. A representative result of three independent experiments is shown. (D) Analysis of cellular interactions between enriched CD56^+^ cells and purified CD14^+^ monocytes. Purified CD14^+^ monocytes were placed in the lower chambers and CD56^+^ cells containing CD56^int^CD11c^+^ cells in the upper chambers. After incubation for 5 days in the presence of IL-2/IL-18, the number of cells in lower chambers was counted and the frequency of cells in the lower chambers was analyzed by flow cytometry. Data show mean ± SD (n = 4), **p<0.01.

As described above, CD56^bright^CD11c^+^ cells were generated in cultures of purified CD56^int^CD11c^+^ cells and CD14^+^ monocytes in the presence of IL-2/IL-18. Stimulation of CD14^+^ monocytes by IL-2, IL-18, or IL-2/IL-18 did not induce the proliferation. Cell division analysis using CFSE-labeled CD14^+^ monocytes also revealed that cellular division was not induced even in the presence of IL-2/IL-18, and the cells appeared to undergo apoptosis in the absence of these cytokines (Fig. 4A).

In order to address the possibility that CD14^+^ monocytes play a critical role in the IL-2/IL-18-mediated development of CD56^bright^CD11c^+^ cells from CD56^int^CD11c^+^ cells, we determined the phenotype of CD14^+^ monocytes. Freshly isolated CD14^+^ monocytes expressed IL-18 receptor α- and β-chains, LFA-1, HLA-ABC, HLA-DR, CD80, CD86, CD40, and CD54 (ICAM-1) (Fig. 4B and 4C). They had enhanced expression of HLA-ABC, HLA-DR, CD80, CD40, CD54, and CD209 after incubation with IL-2/IL-18 for 24h (Fig. 4C). To examine how monocytes are involved in the development of CD56^bright^CD11c^+^ cells from CD56^int^CD11c^+^ cells, transwell tests were performed according to the method described in Material and Methods. To analyze cellular interactions between CD56^int^CD11c^+^ cells and CD14^+^ monocytes, transwell chambers with a pore size of 5 μm for cell penetration tests and those with a pore size of 0.4 μm for soluble factor permeability tests were employed (Fig. 4D). Purified CD14^+^ monocytes (>98% purity) were placed in the lower chambers and CD56^+^ cells (>95% purity) containing CD56^int^CD11c^+^ cells in the upper chambers. After incubation for 5 days in the presence of IL-2/IL-18, the number of cells in lower chambers was counted and the frequency of cells in the lower chambers was analyzed by flow cytometry about CD56 and CD11c expression. Experiments demonstrated that soluble factors alone failed to induce CD56^bright^CD11c^+^ cells from CD56^int^CD11c^+^ cells, and that cell-cell contact between CD14^+^ monocytes and CD56^int^CD11c^+^ cells was critical for the development of CD56^bright^CD11c^+^ cells (Fig. 4D). In accordance with this observation, cell aggregates appeared in co-cultures of CD14^+^ monocytes with CD56^int^CD11c^+^ cells (Fig. 3C, D). Taken together, these results suggest that stimulation of CD14^+^ monocytes by IL-2/IL-18 is essential for the development of CD56^bright^CD11c^+^ cells via intensive cell-cell interactions.

### Inhibition of CD14^+^ monocytes-dependent development of CD56^bright^CD11c^+^ cells by IFN-α

**Figure 5:**
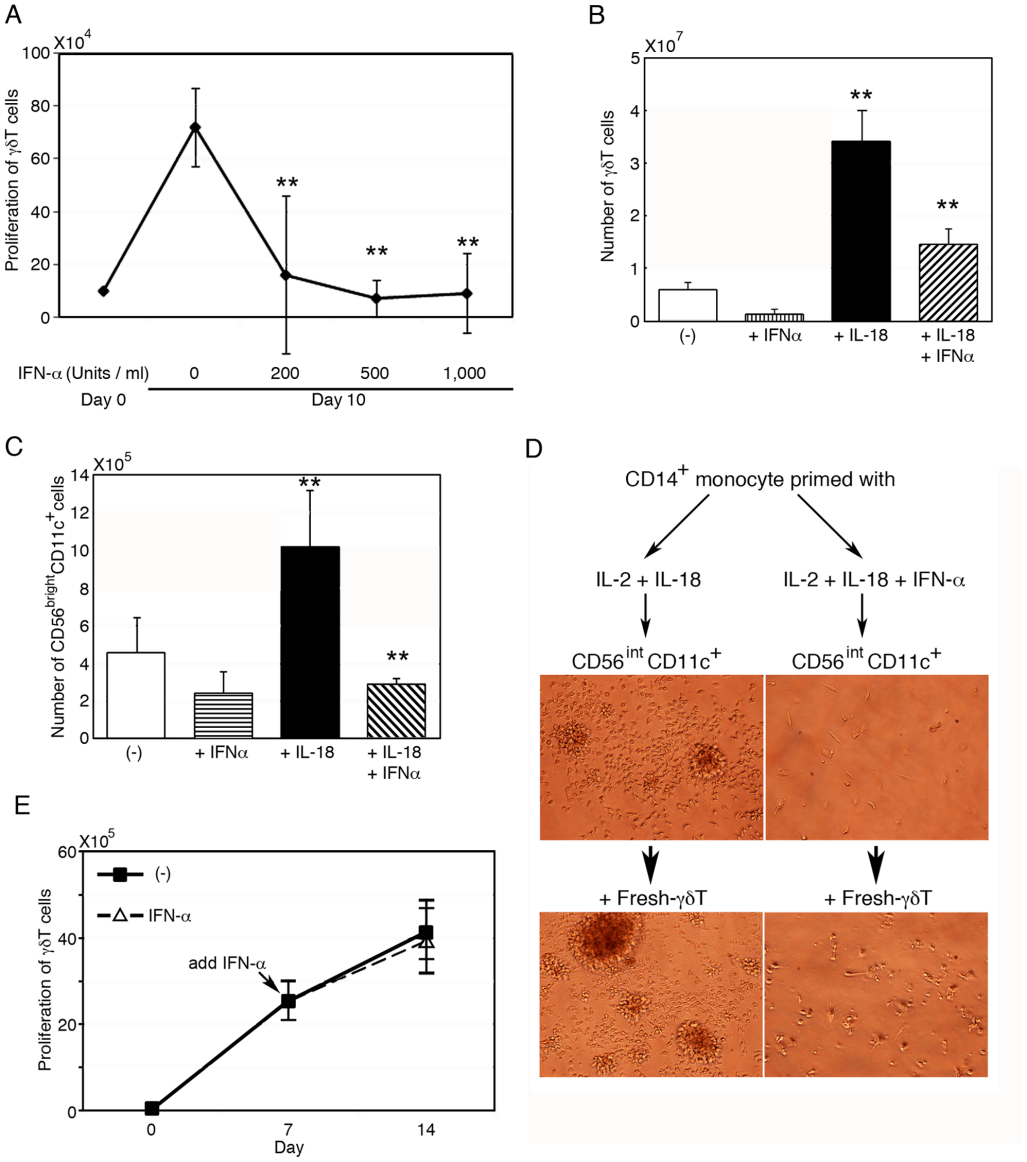
Negative regulation of CD56^bright^CD11c^+^ cell development by IFN-α. (A) Inhibition of γδ T cell proliferation by IFN-α in PBMC cultures. PBMCs were stimulated with ZOL/IL-2 for 10 days in presence of various doses of IFN-α. Data show mean ± SD (n = 5), **p<0.01. (B) Attenuation by IFN-α of IL-18-mediated γδ T cell expansion in PBMC cultures with ZOL/IL-2. Data show mean ± SD (n = 4), **p<0.01. (C) Abrogation by IFN-α of the development and proliferation of CD56^bright^CD11c^+^ cells in cultures of CD3^+^ T cells-depleted PBMCs. Data show mean ± SD (n = 4), **p<0.01. (D) Inhibition of cell aggregation by IFN-α in cultures of CD56^int^CD11c^+^ cells and CD14^+^ monocytes, in the absence and presence of freshly isolated γδ T cells. CD14^+^ monocytes were pretreated with IL-2/IL-18 for 3 days, with or without IFN-α then CD56^int^CD11c^+^ cells were added into the culture (upper panels). Next, freshly isolated γδ T cells were added and cellular clusters were observed by microscope (lower panels). The cell aggregates image is representative of three independent experiments. (E) Proliferation of γδ T cells in cultures containing mature CD56^bright^CD11c^+^ cells even in the presence of IFN-α. Freshly isolated γδ T cells and mature CD56^bright^CD11c^+^ cells were stimulated with ZOL/IL-2, with or without further addition of IFN-α since day 7 onwards and were continuously incubated. The number of proliferating cells was assayed after another 7 days' culture. Data show mean ± SD (n = 5).

Because various cytokines affect differentiation and function of monocytes, we next examined the mechanism by which cytokines could modulate the functions of CD14^+^ monocytes in the development of CD56^bright^CD11c^+^ cells from CD56^int^CD11c^+^ cells. IFN-α is known to facilitate the differentiation of monocytes to IFN-α-DCs that activate CD8^+^ CTLs (35–37). When PBMCs were stimulated with ZOL/IL-2, the proliferation of γδ T cells was abrogated by IFN-α in a dose-dependent manner (Fig. 5A). Whereas IL-18 significantly promoted the expansion of γδ T cells in the culture of PBMCs stimulated by ZOL/IL-2, the addition of IFN-α reduced such expansion (Fig. 5B).

In addition, IFN-α also inhibited the development of CD56^bright^CD11c^+^ cells in cultures of CD3^+^ T cell-depleted PBMCs stimulated with IL-2/IL-18 (Fig. 5C), Furthermore monocytes primed with IL-2/IL-18 in the presence of IFN-α failed to induce generation of CD56^bright^CD11c^+^ cells leading to inhibition of expansion of γδ T cells (Fig. 5D). CD14^+^ monocytes (2×10^5^cells/well) were incubated with or without IFN-α for 3 days in the presence of IL-2/IL-18, then CD56^int^CD11c^+^ cells (2×10^5^ cells/well) were added to the culture. The morphological difference of cells in co-culture was observed by microscopy after additional 3 days (upper panels). Further, freshly isolated γδ T cells (2×10^5^ cells/well) were added to the culture and incubated for another 3 days. Large clusters were observed in the culture incubated with IL-2/IL-18 alone, but not in the culture added with IFN-α (lower panels). Thus IFN-α was suggested to alter the effect of IL-2/IL-18 on CD14^+^ monocytes resulting in inhibition of development of CD56^int^CD11c^+^ cells to CD56^bright^CD11c^+^ cells.

Furthermore, it is of note that the expansion of γδ T cells was not diminished by the addition of IFN-α when freshly isolated γδ T cells were co-incubated with mature CD56^bright^CD11c^+^ cells (Fig. 5E). Freshly isolated γδ T cells and mature CD56^bright^CD11c^+^ cells (purified from IL-2/IL-18-pretreated CD3^+^ depleted PBMCs for 7 days) were co-cultured with ZOL/IL-2, with or without further addition of IFN-α since day 7 onwards and were continuously incubated. The number of proliferating cells was assayed after another 7 days' culture. Proliferation of γδ T cells appeared not to be influenced by IFN-α. These results suggest that IFN-α abrogated the expansion of γδ T cells by inhibiting the IL-2/IL-18-mediated development of mature CD56^bright^CD11c^+^ cells from CD56^int^CD11c^+^ cells, rather than directly inhibiting the growth of γδ T cells.

## Discussion

Evidence is accumulating that human γδ T cells are involved in the first line of defense against infections and malignancies. Although much attention has been paid to cancer immunotherapy using γδ T cells, hyporesponsiveness of γδ T cells to phosphoantigens has hampered the development of novel γδ T cell therapy for cancer patients. Thus, it is important to clarify the cellular mechanisms underlying the expansion of γδ T cells.

**Figure 6:**
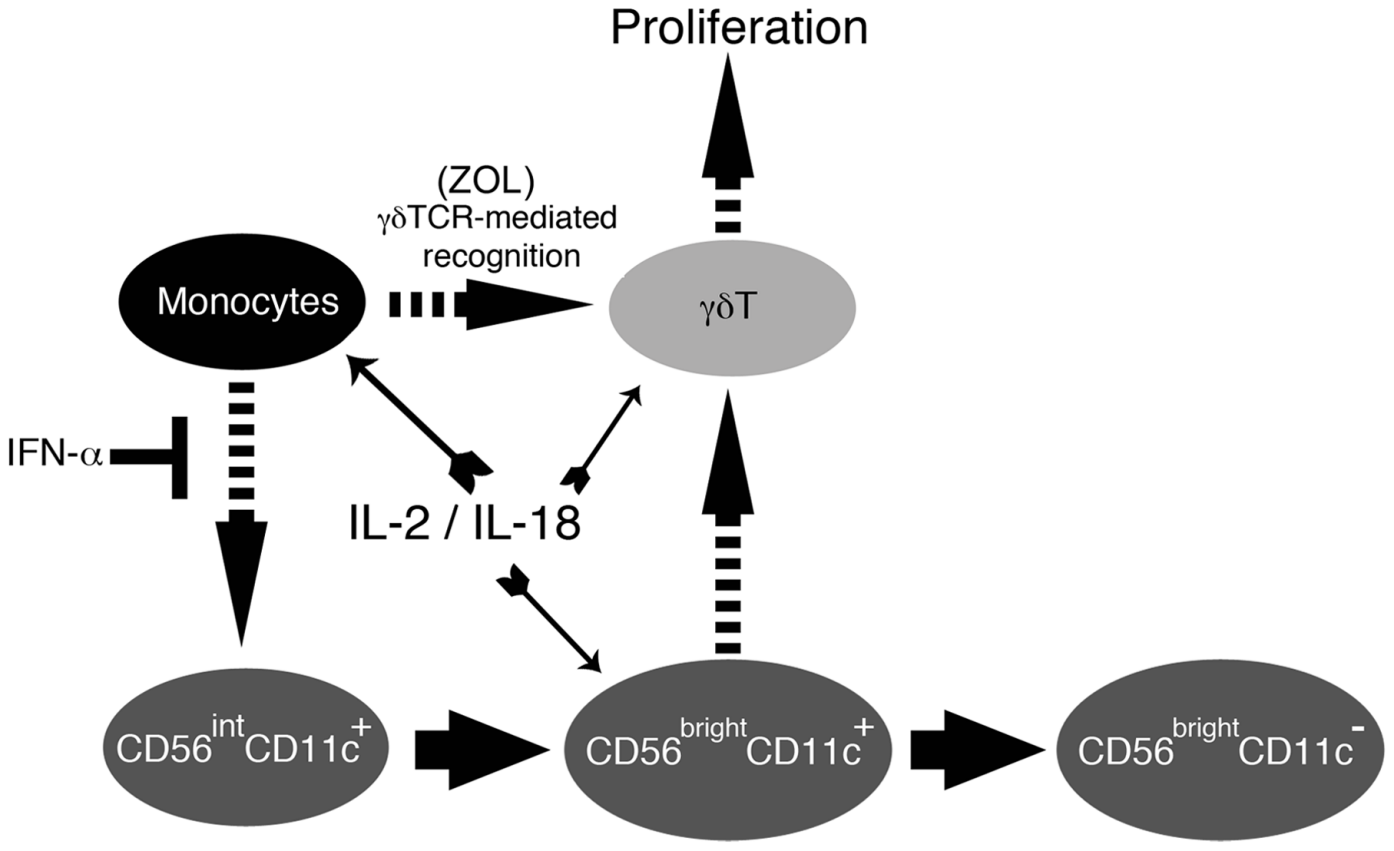
Putative model for the regulation of the development and expansion of CD56^bright^CD11c^+^ cells and γδ T cells. In response to ZOL, CD14^+^ monocytes stimulate γδ T cells in a TCR-dependent manner. Concomitantly, CD14^+^ monocytes induce the IL-2/IL-18-mediated generation of CD56^bright^CD11c^+^ cells from their putative precursor CD56^int^CD11c^+^ cells. IFN-α inhibits this process possibly through the production of IFN-α-DCs. The resulting CD56^bright^CD11c^+^ cells initiate and promote the expansion of γδ T cells for several days and gradually lose their helper function as they lose CD11c expression.

CD14^+^ monocytes can internalize nitrogen-containing bisphosphonates (N-BPs) such as ZOL and present IPP or IPP-related antigens to γδ T cells. This fluid-phase endocytosis occurs predominantly in CD14^+^ monocytes at relatively low concentrations of N-BPs. Thus, CD14^+^ monocytes play an essential role in antigen presentation to γδ T cells. Whereas CD14^+^ monocytes are pivotal in the initial activation of γδ T cells, they fail to directly support the subsequent proliferation of γδ T cells. The present study revealed that CD14^+^ monocytes not only functioned as APCs, but also were important for the development and proliferation of CD56^bright^CD11c^+^ cells that could directly act on γδ T cells to positively regulate their expansion (Fig. 6).

Because CD56^bright^CD11c^+^ cells were generated in cultures of CD56^int^CD11c^+^ cells and CD14^+^ monocytes in the presence of IL-2/IL-18, it was likely that CD14^+^ monocytes induced the generation of CD56^bright^CD11c^+^ cells from CD56^int^CD11c^+^ cells. Treatment of CD14^+^ monocytes with ZOL inhibits, farnesyl diphosphate synthase, an enzyme in the isoprenoid pathway involved in the biosynthesis of isoprenoid metabolites, causing a low intracellular level of geranylgeraniol, which may be responsible for the activation of caspase 1 and the maturation of IL-18 [13]–[18]. Endogenous IL-18 is thus essential for the development of CD56^bright^CD11c^+^ cells, while other soluble factors or signaling pathways required for interactions between CD14^+^ monocytes and CD56^int^CD11c^+^ cells remains to be identified.

Nascent CD56^bright^CD11c^+^ cells developed under the influence of IL-2/IL-18 expressed an intermediate level of CD11c, a high level of APC-related molecules such as CD80, CD86, HLA-DQ, and HLA-DR, and a high level of ICOS and CD25 [24], [25]. These cells tended to aggregate among themselves without exogenous cytokines or chemokines. When they were mixed with γδ T cells, they quickly formed large cell aggregates and an efficient proliferation of γδ T cells was observed even in the absence of antigens. None of the other cell types, including monocytes, conventional DCs, IFN-α-induced DCs, and pDCs formed cell aggregates with γδ T cells. This indicates that nascent CD56^bright^CD11c^+^ cells have a natural affinity to γδ T cells and promote their proliferation in an antigen-independent manner. However, proliferation may be adhesion molecule-dependent because both cell types express adhesion molecules such as LFA-1 and ICAM-1. The addition of ZOL further enhanced the proliferation of γδ T cells demonstrating that CD56^bright^CD11c^+^ cells can also serve as APCs, while the efficiency for fluid-phase endocytosis of ZOL might be lower than that of CD14^+^ monocytes.

During the course of cell culture, CD56^bright^CD11c^+^ cells tended to concomitantly lose CD11c expression and the ability to support γδ T cell expansion. The supporting function of CD56^bright^CD11c^+^ cells reached a peak between 3 and 7 days after the start of cell culture. When CD11c expression was completely lost between day 15 and day 21, they also lost their supporting function. CD11c by itself, however, might not be involved in the helper function, because other CD11c-highly-expressing cells failed to both form cell aggregates and enhance γδ T cell proliferation.

Consistent with our previous reports, CD56^bright^CD11c^+^ cells showed a high level of tumoricidal activity in an effector-to-target ratio-dependent manner. In this study, we further examined the specificity of the cells. It is of note that CD56^bright^CD11c^+^ cells could recognize and lyse malignant tumor cells, distinguishing them from normal cells. This finding provides compelling evidence to support the classification of CD56^bright^CD11c^+^ cells as NK-lineage cells. Because both NK-lineage cells and γδ T cells belong to innate immune cells, they may interact and serve as a bridge between the innate and adaptive immune responses. Indeed, both CD56^bright^CD11c^+^ cells and γδ T cells express high levels of APC-related molecules and may present peptide antigens derived from lysed tumors and infected cells to conventional αβ T cells.

This study demonstrated that CD14^+^ monocytes could induce the development of NK-like CD56^bright^CD11c^+^ cells under the influence of IL-2/IL-18 and positively regulate the proliferation of γδ T cells by an indirect mechanism. Because many reports have suggested interactions between NK cells and monocytes/DCs, which may determine the differentiation and proliferation of distinct subsets of NK cells, we next attempted to determine the molecular mechanism that negatively modulated the development of CD56^bright^CD11c^+^ cells and subsequently impaired γδ T cell expansion.

We demonstrated that cell-cell contact was important for the development of CD56^bright^CD11c^+^ cells. Neither purified CD14^+^ cells nor CD56^int^ CD11c^+^ cells alone expanded to CD56^bright^CD11c^+^ cells. Therefore, not only soluble factors such as cytokine, and cellular interaction between CD14^+^ cells and CD56^int^ CD11c^+^cells were necessary for CD56^bright^CD11c^+^ cells generation, as confirmed by transwell and co-culture tests. IL-18 prolonged the viability of CD14^+^ monocytes, and up-regulated the expression of co-stimulatory molecules such as HLA-ABC, HLA-DR, CD80, CD40, and ICAM. Because freshly isolated CD14^+^ monocytes expressed both Rα and Rβ chains of IL-18R, IL-18 may directly transduce signals to monocytes. Recently, M-CSF-stimulated monocytes were shown to express membrane-bound IL-18 [41], [42], although the physiological roles and functions of these monocytes in inflammation remain to be explored. It may be of interest to examine the relationship between IL-18-stimulated monocytes and IL-18-expressing monocytes. Further study is required to clarify the development, phenotypes, and roles of these monocytes.

It is generally difficult to expand γδ T cells from PBMCs that contain a low frequency of γδ T cells, compared with PBMCs that contain a high frequency of γδ T cells. When PBMCs with a low γδ T cell frequency are stimulated with ZOL/IL-2, many adherent cells can be seen by microscope. In contrast, only large cell aggregates are present when PBMCs with a high γδ T cell frequency are used. This suggests that to expand subsets of effector cells the corresponding APCs are necessary as supporting cell types. For example, conventional DCs and IFN-α-DCs support the development of CD8^+^ CTL cells, and PGE-2-DCs plays role in the augmentation of regulatory T cells. In the case of PBMCs with a low γδ T cell frequency, pDCs survived and inhibited the generation of CD56^bright^CD11c^+^ cells, leading to the impaired proliferation of γδ T cells (data not shown). Taken together, this suggests that IL-18 may activate CD14^+^ monocytes to facilitate the expansion of both CD56^bright^CD11c^+^ cells and γδ T cells. Furthermore, it is worth noting that IFN-α failed to interfere with the functions of mature CD56^bright^CD11c^+^ cells, because the addition of mature CD56^bright^CD11c^+^ cells to the in vitro culture system overcame the inhibitory effect of IFN-α.

Although IL-18 was originally discovered as an IFN-γ-inducing factor, its physiological roles have not been fully clarified. The present study demonstrated that IL-2/IL-18 facilitated the development of CD56^bright^CD11c^+^ cells by CD14^+^ monocytes. Because activated γδ T cells also express IL-18 receptor α and β chains, IL-18 may directly act on γδ T cells. In addition, IL-18 has been shown to maximize innate immune responses of ITAM-bearing lymphocytes and up-regulate Bcl-2 and Bcl-X_L_, which protect mitochondria and augment survival signaling. IL-18 was recently shown to prime NK cells to have unique helper activity, and the resulting “helper” cells promote activation of DC and DC-mediated recruitment of effector CD8^+^ T cells to the tumor microenvironment [43]. It is thus intriguing to compare the physiological function and roles of “helper” NK cells with the present CD56^bright^CD11c^+^ cells as well as murine IKDCs or NKDCs.

Based on the present results, signals transduced from TCR, co-stimulatory receptors, adhesion molecules, and IL-18 receptors are required for the full activation and sustained proliferation of γδ T cells. In conclusion, CD14^+^ monocytes play a critical role in the generation of novel CD56^bright^CD11c^+^ cells that directly interact with activated γδ T cells and sustain their robust expansion. This function of monocytes can be altered by IFN-α, which induces the differentiation of CD14^+^ monocytes to IFN-DCs (Fig. 6). The precise, physiological role of IL-18, CD14^+^ monocytes, and CD56^bright^CD11c^+^ cells in innate immune responses remains to be established.
